# Filling Teeth over Exposed Nerves

**Published:** 1853-01

**Authors:** S. P. Miller

**Affiliations:** Worcester, Mass.


### *Filling Teeth over Exposed Nerves.*—By S. P. Miller, *of Worcester, Mass.*

No subject in operative dentistry has more earnestly engaged the attention of members of the dental profession, for a series of years, than filling teeth after their nerves have become exposed. This is owing to the frequency of the occurrence and the uncertain results which flow from it. That the teeth exert an important influence over the animal economy—that their appropriate functions are necessary to a healthy nutrition and assimilation—that a disturbance of these functions leads to a derangement, more or less, of parts intimately associated with and dependent upon them—that their preservation is requisite to a perfect intonation and modulation of the human voice, are physiological facts which need no argument to sustain them.

So marked is the sympathetic influence of diseased teeth upon adjacent organs, and parts more remote, that it is universally conceded point among well-informed dentists, that he who can most effectually counteract it and restore their impaired functions, confers a larger benefit upon the community than the merely *mechanical* dentist; and that it is the duty, therefore, of every operative dentist, in the exercise of his professional skill, to study carefully, both the physiology and pathology of these important organs, in order that, to their various forms of disease, he may be able to make appropriate applications. Formerly, by some of the most eminent physiologists, the teeth were considered as isolated bodies, having little connection or sympathy with the general system; but modern investigation, and the improved methods of treatment, have elevated the science of dentistry, in the true sense of the term, to a speciality in medicine.

The operation of filling teeth when there is little or no sensibility, is mainly mechanical, and may be done by persons of fair mechanical tact who have not received a medical education; but when their sensibility becomes exalted, or their lining membranes exposed—when their diseases become complicated with those of other tissues, compromising the general welfare, it becomes a matter of deep interest to the patient, and calls for discrimination and decided action on the part of the operator.

Much has been written upon the subject of exposed nerves, and various modes of treatment have, from time to time, been resorted to, such as the application of astringents, caustics, the use of essential oils, destroying them with arsenic, &c,—capping them with lead, tin, or gold plate; but all of these have been tried with ill success, in numerous instances, greatly to the discomfort of patients, and the vexation and disappointment of dentists. Nevertheless, to meet the desires of their patrons, dentists are often obliged to adopt some one or more of these modes, with little hope of ultimate success.

Arsenic, although the most efficient and certain means of removing the sensibility, or destroying the nerves of teeth, is not free from objections, in that it seems not well adapted to certain temperaments, being sometimes absorbed by the dentine and the surrounding tissues, producing an inflammation, extending to the peridental membrane—causing a slough of the gum, an exfoliation of the alveolar process, and the removal of the offending tooth. Such results, though seldom, have been known to take place where arsenic had been employed even in small quantities, but may have been owing, in part, however, to the difficulty of retaining it within the cavity. So weighty are these objections, in the minds of some dentists of acknowledged reputation to its employment, whether alone or in combination with anodynes, that they selom use it in their practice

The absorption of arsenic, producing death of the nerve, leaves a *poisoned* wound, with a bloody discharge, which must be healed, and anastomosing vessels formed, before the tooth can be filled, with even *comparative* safety.

But another and somewhat serious objection to the use of arsenic, lies in the fact that the use of the teeth, which have been filled after the destruction of their nerves often become diseased from morbid effects produced by it, requiring extraction within a few months, or, at farthest, a year or two, notwithstanding the filling remain sound. In some instances, too, after the loss of vitality from the use of arsenic, the teeth become discolored, presenting an *extravasated* appearance, which, in a *frontal denture*, greatly mars its beauty.

With the idea of remedying these defects to some extent, of substituting a *healthy* for a poisoned wound, of retaining, to the fullest degree possible, the vitality, life-like appearance, and unimpaired functions of the teeth, nearly two and a half years ago I instituted a surgical operation which had been in contemplation for several months.

CASE 1. June 5th, 1850.—The left superior central incisor tooth of Miss H., and the modus operandi as follows. Having wounded the nerve in preparing the cavity for filling, about an eighth of an inch from the margin of the gum, with a small, sharp excavator I made a straight puncture through the alveolus to the fang directly opposite its centre; then, with a drill about the size of the nerve, I drilled through the fang to the nerve, which in this case, being small, was entirely amputated. It was the intention to amputate the nerve with a suitable instrument, in case it were not done by the drill. All sensibility between the opening and the pulp being cut off, the tooth was filled in the usual manner and without pain. It is important that the puncture be made directly over the centre of the tooth and *through* the alveolar, to serve as a guide to the drill, otherwise the object may be defeated without enlarging the opening more than is necessary. The drill should be worked slowly, removed often and the point dipped in water to prevent its becoming heated; if not, it will cause pain and slight inflammation. The head of the drill should be about the size of the nerve, if amputation be contemplated, a little larger than the shaft, in order that the bone-dust or drillings may escape freely, flattened, and the point sharp and properly tempered. The shaft should have its temper drawn nearly to the head, so that should the patient start suddenly, it may bend rather than break. For this operation one of Babbett's spiral drill-stocks is far preferable to the bow, it being more firmly held by the operator and less likely to slip. After drilling, the bone-dust should be entirely removed from the wound, otherwise it will not cicatrize readily.

October 1st, nearly four months after the operation, the patient called again to have other teeth filled, and reported that there had been no pain, nor but little soreness, and *that* where the gum was punctured. An examination proved what was anticipated at the time of the operation, viz., a re-union of the divided nerve, showing that the recuperative energy of the nervous system exists, as well in the teeth as in other organs. Examinations have shown, too, that the nerve sometimes becomes ossified so as to prevent the introduction of a small instrument into the canal through the opening made by the drill. This tooth presented a natural, healthy appearance, free from discoloration, and in all respects was as serviceable as before it was filled.

Case II. June 6th.—J. C. Teeth operated on—the right superior canine and two bicuspids. On finding their nerves exposed, and having amputated the nerve of the cuspidatus, a query arose as to what should be done with the bicuspids having *two* nerves. After a moment's reflection, the drill was carried deeper, cutting off both branches, and the teeth filled without pain. The patient was requested to call and report the case, which he did the third day after, by saying—"all right." On examinatoin, the teeth and gum presented a healthy appearance, the cicatrices being scarcely visible. He promised to inform me if the operation did not prove successful, but has not been heard from.

Having treated a few cases in this way, I determined to go a step farther—after amputation to remove the pulp from the nerve cavity. This was done in several instances, the result of which in every case, so far as known, has been as successful as when the pulp was allowed to remain. In cases where the pulp is removed, the teeth are not sensible to impressions from heat or cold.

But the inquiry extended still farther—to the molar teeth having two, three, and sometimes four branches of nerves, and situated so far back in the mouth as to be difficult of access. It was readily seen that this class of teeth, from their position and the number of their fangs, each having a nerve, could not be subjects of this operation as performed on the other teeth. This suggested another experiment—that of drilling into the nerve cavity under the festoon of the gun, wounding the pulp as little as may be, then to cover the exposed part with a pellet of gold made flat and hard, so as to prevent pressure, and leave the result to the *vis medicatrix naturæ*, and for whatever treatment the case might require. This was attended with a result satisfactory to the patent; all that was done being to penetrate the chamber of the tooth to serve as an outlet for the escape of pus, should inflammation and suppuration intervene.

Having treated a molar tooth successfully, and besides amputation of the nerve, either with or without removal of the pulp, especially in bicuspids with two branches, in some instances requiring considerable time, and being somewhat painful to the patient and difficult for the operator, it occurred that drilling a little nearer the margin of the gum, so as, in a bicuspid, to strike the outer branch near the pulp, slightly wounding it, would effect the desired object.

This operation being more expeditiously performed, and with much less pain to the patient, since its successful employment cutting off the nerve has been practiced in but few instances. After having operated in about forty cases, embracing the different classes of teeth, with results far better than had been anticipated, without the loss of a tooth, so far as known, some six months after my first experiment, I communicated the idea and method of operating to a few friends, with the request that they would carry it out in their practice, and report their success. This several of them have done, and so fully does it correspond to my own, as to challenge a comparison between this and any other known method of filling teeth after their lining membranes become exposed.

There are instances in which amputation may be resorted to in filling teeth when their nerves are *not* exposed. Every operative dentist is familiar with cases where the teeth, for instance, the incisor and canine, are superficially decayed, but whose sensibility is too much exalted to allow of the removal of the caries preparatory to filling, unless it be in some way reduced. The common mode of doing this is to apply a little arsenic and cover it with wax, and repeat the operation till the object be accomplished.

A case of this kind occurred Feb. 13th, of the current year, in a lady having six upper and three lower front teeth to be filled. In all of them the sensibility being too acute to admit of the operation, chloroform was administered and three of the nerves cut, after which the teeth were filled without pain. Eight days after, Feb. 21, three others were served in the same way, and on the 28th of the same month the remainder were filled. April 22d, several of the same teeth required filling in other places, when it was found that the nerves were re-united and the sensibility returned, though not in a degree to require a second amputation. In this case, for several weeks there was considerable soreness and slight tumefaction of the gum about the place of puncture, as not unfrequently happens in strumous habits. The patient remarked that she had a "scrofulous temperament, and that a wound of any kind was a great while in getting well."

Whenever the gum assumes a fungoid appearance, nitrate of silver may be applied with benefit. It has been observed that there is less swelling and soreness in those cases where the opening was made under the festoon or margin of the gum, than when made through it, or by raising a small flap before drilling.

Having subjected between two and three hundred teeth to various experiments, with the loss of one tooth only (the inferior right second bicuspid), the method which I prefer and generally practice, is, to insert the drill under the edge of the gum where the enamel terminates, and barely make an opening *to* the nerve (with a smaller drill than is used for amputating) wounding it as slightly as possible; then to protect the exposed nerve from pressure, and plug the tooth in the usual manner.

Without going further into detail, I herewith submit the subject to the medical and dental profession, trusting that it may receive a thorough and candid investigation.—*Bost. Med. and Sur. Jour.*

